# Retroperitoneal Necrosis as a Rare Complication After Celiac Plexus Block

**DOI:** 10.7759/cureus.13169

**Published:** 2021-02-06

**Authors:** Ya Zhou, Brendan O'Donovan, Jorind Beqari, Reginald Alouidor

**Affiliations:** 1 Surgery, University of Massachusetts Medical School-Baystate, Springfield, USA; 2 Anesthesiology, University of Massachusetts Medical School-Baystate, Springfield, USA

**Keywords:** celiac block, complications, pain management

## Abstract

Long after surgical treatment, chronic pain continues to afflict many patients with pancreatic cancer. Multimodal pain management is the current approach to managing these complex patients. In patients with refractory pain, a celiac plexus block is a commonly used adjunct to optimize pain control. The sclerosing agents used in a celiac plexus block are known to cause local tissue necrosis as a rare complication. We present a case of extensive retroperitoneal necrosis following celiac plexus neurolysis. To our knowledge, this is the first report of extensive retroperitoneal necrosis after a celiac plexus block requiring operative management.

## Introduction

A celiac plexus block is indicated for the treatment of intractable abdominal pain in the setting of malignant and benign neoplasms involving the pancreas, the biliary tree, the retroperitoneum, and other abdominal organs. The most common indication is for patients with pancreatic cancer. Many patients with pancreatic cancer have debilitating chronic pain that is difficult to manage with medication alone. Celiac plexus block is an adjunct to oral analgesics, which involves the injection of steroids, long-acting local anesthetic agents, epinephrine, clonidine, and other agents to the celiac plexus. Neurolysis with alcohol or phenol agents has a more permanent effect. Complications from celiac plexus block are rare with the most common being diarrhea and orthostatic hypotension. This is reported in 34% and 44% of patients respectively [1]. There have been reports of more serious complications including end-organ ischemia in the distribution of the celiac artery secondary to celiac plexus neurolysis [2]. Here, we describe a case of a 29-year-old male status post distal pancreatectomy and splenectomy for extraosseous Ewing’s sarcoma of the pancreas treated with celiac plexus neurolysis who presented acutely with extensive retroperitoneal soft tissue necrosis.

The celiac plexus is located at the level of L1, anterior to the aorta and posterior to the pancreas. The greater, lesser, and least splanchnic nerves, arising from T5 to T12, provide the preganglionic innervation to the celiac plexus. Postganglionic fibers leave the celiac plexus and innervate the embryonic foregut [3].

There are different techniques to perform neurolysis of the celiac plexus. The “classic technique” is the retrocrural technique. With the patient positioned prone, a needle is directed just lateral to the vertebral body and is gradually advanced until pulsations from the aorta are felt. For a diagnostic or prognostic block, a local anesthetic such as lidocaine is used. For therapeutic neurolysis, 10-25 cc of 50% ethyl alcohol or 6.0% aqueous phenol is used.

Image-guided trans-aortic celiac plexus block is now being recognized as a safer alternative to the classic retrocrural technique. The patient is again positioned prone and a scout film is used to determine the level of T12-L1 interspace and to review any distorted anatomy that may be due to malignancy or previous surgery. A stylet needle is advanced until the posterior wall of the aorta is encountered. The stylet is removed and the needle hub will show arterial blood. A saline-filled glass syringe is attached and the needle is advanced using a loss-of-resistance technique. Contrast medium is injected once the loss of resistance occurs and a CT scan is taken and reviewed for proper needle placement and spread of contrast. Once proper placement in the pre-aortic space is ensured, the neurolytic agent is injected.

## Case presentation

Our patient is an otherwise healthy 29-year-old who had been previously diagnosed with Ewing’s sarcoma of the pancreas and underwent a distal pancreatectomy with splenectomy. He had undergone 10 cycles of vincristine, doxorubicin, cyclophosphamide alternating with ifosfamide, etoposide (VDC/IE) chemotherapy. He had significant postoperative abdominal pain, nausea, and vomiting refractory to oral pain agents. Chronic constipation also likely contributed to his symptoms. Due to persistent pain, the patient was referred for celiac plexus block.

A trans-aortic L1 celiac plexus block was performed under fluoroscopic guidance. 5 cc of 2% lidocaine with 80 mg of methylprednisolone was injected into the pre-aortic space, which was followed by 30 cc of 50% alcohol. There were no immediate post-procedural complications, and the patient was discharged home with a short course of oral opioids, anti-emetics, and a bowel regimen.

**Figure 1:**
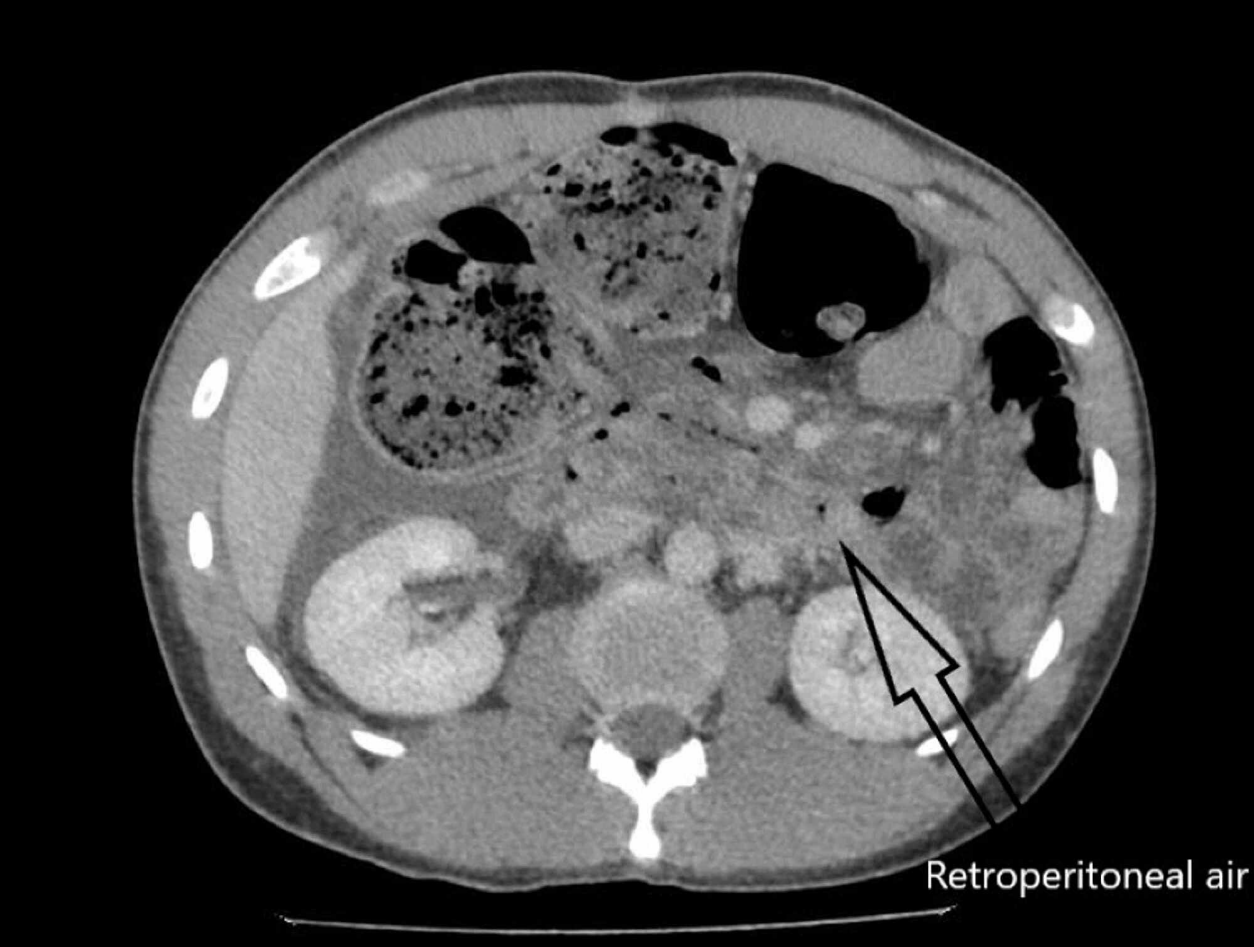
Axial section of CT showing retroperitoneal air

Two days later, the patient returned to a local hospital with severe abdominal pain and was found to have peritonitis on exam. The initial workup was significant for leukocytosis, and CT imaging showed retroperitoneal free air with suspicion of retroperitoneal duodenal perforation (Figure 1). The patient was transferred to our hospital for further care. The patient was urgently brought to the operating room for exploration due to his septic state and peritonitis. His vital signs were significant for tachycardia but he was otherwise normotensive and afebrile. We proceeded with an exploratory laparotomy and found areas of necrosis in the right retroperitoneum. There was also extensive necrotic tissue involving the serosa of the duodenum along the 2nd and 3rd portions as well as Gerota's fascia of the right kidney. The mesentery of the right colon also appeared necrotic, but the bowel itself appeared viable. We surmised that the free air seen on imaging may have been from a small contained duodenal perforation. We therefore proceeded with a damage control temporary abdominal closure with a planned second look. He returned to the operating room within 24 hours. Minimal residual necrotic tissue of the retroperitoneum remained, and a drain was placed near the duodenum prior to formal abdominal closure.

His postoperative course was complicated by prolonged ileus and a low-volume pancreatic leak well controlled with the operative drain. He had persistent leukocytosis postoperatively, which was most likely from the residual necrotic tissue, and resolved by discharge. There were no signs of ongoing sepsis. The patient was evaluated by the acute pain service and started on high-dose morphine patient-controlled analgesia (PCA) that was transitioned to oral morphine and slowly tapered over the course of his hospitalization. The patient was eventually started on a diet with continued low output from the pancreatic leak. He was discharged home on hospital day 24 and instructed to follow up with his medical oncologist for chronic pain management. 

## Discussion

Chronic pain is a well-established clinical challenge in patients with pancreatic malignancy. Patients often require long-term pain medication regimen, including adjunct analgesic procedures such as the celiac plexus block. Pain due to pancreatic malignancy was proposed by Lahoud et al. to be a combination of three physiologic pathways: visceral, somatic, and neuropathic [4], each of which is specifically targeted by available treatment modalities. Medical approaches include a step-up approach of non-narcotic analgesics to mild opiates then to stronger opiates. Antiepileptics such as pregabalin and gabapentin are also utilized, especially in the presence of neuropathic pain. Lastly, corticosteroids have been shown to improve visceral pain by inhibiting prostaglandin and the subsequent inflammatory cascade. In addition to medical therapy, chemoradiation is another treatment modality for refractory pain. Gemcitabine has been approved for pancreatic cancer since 1997 for both treatments of advanced disease and pain control [5].

Similarly, 5-fluorouracil, leucovorin, irinotecan, and oxaliplatin (FOLFIRINOX) showed improved pain control and quality of life in metastatic pancreatic cancer patients [6]. Radiation therapy serves multiple purposes in pancreatic cancer, in the adjuvant setting for disease control as well as for pain control in large tumors that compress into surrounding structures [4]. Often, the pain remains poorly controlled and other modalities have been explored, including interventional therapies and alternative medicine.

The celiac plexus block and neurolysis are used as adjuncts to medical regimens when patients still experience significant pain. Common complications include hypotension and diarrhea. Hypotension occurs due to regional vasodilation. This can be mitigated by initiating a 500 cc to 1 liter bolus of crystalloids prior to the procedure. Self-limited diarrhea occurs due to unopposed parasympathetic activity after the block.

Additional severe complications have been reported, including intravascular injection, epidural or subarachnoid injection, renal injury, inadvertent neurolysis of the lumbar somatic nerves, peritonitis, abscess, retroperitoneal hematoma, injection of the psoas muscle, pneumothorax, bilateral diaphragmatic paralysis, and ejaculatory failure [7,8]. There have been isolated case reports of organ ischemia following celiac plexus block/neurolysis, including one case of splenic necrosis [9]. These severe complications are rare and are mostly cited as case reports in the literature.

In the early 1990s, a retrospective study looked at the rates of permanent paraplegia following celiac plexus block in England and Wales during the years 1986-1990. There was a total number of four cases of paraplegia out of 2730 blocks (0.14%) that were performed over those years [10]. In 2009, a retrospective study looked at the rates of complications from endoscopic ultrasound-guided celiac plexus block. In the study, 189 patients had celiac plexus blockade without neurolysis and three patients had a complication. Complications included retroperitoneal abscess in one patient and severe self-limited post-procedural pain in two patients [11].

Optimizing pain control in patients with pancreatic malignancy continues to be a complex and elusive moving target. Adjuncts to oral analgesics are frequently required, including celiac plexus block/neurolysis. The use of sclerosing agents likely causes local inflammation and necrosis which remains clinically occult most of the time. However, our case presentation is one example of a severe complication that can be better understood with future studies on this commonly performed procedure.

## Conclusions

Our patient experienced a rare case of extensive retroperitoneal necrosis necessitating operative intervention after a celiac plexus block. As a frequently used adjunctive pain control modality in palliative care, the celiac plexus block warrants additional research to ensure safety and adequate post-procedural monitoring.
